# Similarities in socioeconomic disparities and inequalities in women’s nutritional status and health care in Bangladesh, Ethiopia, India, and Nigeria

**DOI:** 10.1080/16549716.2024.2439165

**Published:** 2025-01-17

**Authors:** Tina G. Sanghvi, Edward A. Frongillo

**Affiliations:** a Alive & Thrive, FHI 360, Washington, USA; b Department of Health Promotion, Education, and Behavior, University of South Carolina, Columbia, SC, USA

**Keywords:** Socioeconomic status, disparities affecting women, prenatal supplements, women’s body mass index, maternal health care access, Asia

## Abstract

Background: Reducing inequalities in women’s nutrition and health care can accelerate progress towards Sustainable Development Goals for maternal and child health. Nutrition interventions for women are delivered through maternal health services such as antenatal care and institutional deliveries, but whether they reach and protect the disadvantaged against malnutrition is not well documented. Objective: To assess the similarities in socioeconomic disparities and inequalities in the nutritional status and health care of women. Methods: We analyzed nationally representative data from Demographic and Health Surveys (DHS) conducted in Bangladesh, Ethiopia, India, and Nigeria to calculate Erreygers index. This index measures the inequality in outcomes across socioeconomic spectrums. We investigated inequalities in low and high body mass index (BMI), anaemia, iron and folic acid supplementation, four or more antenatal care visits, institutional deliveries, and access to health services. Results: Anaemia (−0.068 to −0.123), low BMI (−0.088 to −0.139), perceived distance to health services (−0.219 to −0.406), and needing permission to visit health facilities (−0.062 to −0.147) were concentrated among the less well-off, as shown by negative values. Iron and folic acid supplementation (0.043 to 0.230), antenatal care visits (0.260 to 0.495), and institutional deliveries (0.168 to 0.573) favored the better-off, as shown by positive values. Inequalities in urban vs. rural areas differed by indicator and country. Conclusions: Nutritional status and health care inequalities among women followed patterns of socioeconomic disparities. Inequalities in nutritional status favored the better-off and educated women, and inequalities favoring the better-off were even greater for health care across countries.

## Paper context

- **Main findings**: Higher maternal undernutrition and poor health care among women from socioeconomically disadvantaged households are well known. Little is known, however, about whether health services that deliver nutrition interventions have similar patterns of inequalities, and if there are differences across countries and urban/rural areas.
- **Added knowledge**: This work presents evidence of similarities in patterns of socioeconomic disparities and inequalities in maternal nutritional status and access to health care and identifies country differences.
- **Global health impact for policy and action**: Variations in inequalities across and within countries in over- and under-nutrition, health coverage, and access show that country investments are urgently needed to implement geographically targeted health care services and nutrition interventions in antenatal care for women from marginalized households.

## Background

Poor nutrition and dietary practices contribute to the lack of progress in women’s health outcomes, well-being, and productivity [1–3]. Low body mass index (BMI) or thinness, high BMI or overweight and obesity, and anaemia are common among women of reproductive age (15–45 years) particularly in low- and middle-income countries (LMICs) due to insufficient or unhealthy diets, frequent illnesses, sedentary lifestyles, or a combination of these factors, and they can influence work productivity, mental health, and the risk of maternal morbidity, mortality and poor birth outcomes [2]. According to the Global Burden of Disease analysis, poor health outcomes attributable to dietary risks were 11 million (range 10–12) deaths and 255 million (range 234–274) DALYs in 2017 [4].

Nutrition interventions include improving knowledge and food access to achieve adequate dietary intakes, promotion of balanced energy consumption and physical activity, and provision of supplements and counseling on adherence [5–7]. Through the provision of nutrition counselling and education, and sometimes cooking demonstrations, families can be empowered to source and prepare nutritious meals. Understanding the nature and magnitude of socioeconomic inequalities in nutritional status and – since nutrition interventions for women are mostly delivered through maternal health services – reducing inequalities in health care coverage among women are important steps towards improving women’s nutrition and health outcomes. According to WHO, to realize the benefits of quality health care, health services must be equitable, benefit those most in need, and be provided in an integrated and efficient way [8].

Most LMICs are facing the triple burden of malnutrition among women, i.e. undernutrition, overweight/obesity (which in turn is associated with non-communicable diseases, NCDs), and micronutrient deficiencies (2, 3) including anaemia (4, 5). Country and rural/urban differences in social, ecological, and economic conditions affect women’s nutritional status, dietary practices, and health care utilization resulting in diverse outcomes because of community norms regarding food taboos in pregnancy, gender bias against women, and fluctuations in the production and prices of nutrient-rich foods, are among the determinants of women’s diet and nutrition adequacy [9–11]. Reductions in inequalities are expected to accelerate progress in Sustainable Development Goals 2.2 (maternal and child nutrition) and 3.1 (maternal mortality rates) [12]. This expected acceleration is related to greater risk of adverse outcomes among socioeconomically disadvantaged women due to low nutrition reserves and high exposure to disease leading to conditions such as anemia and hypertensive disorders, and adverse outcomes among their newborns and infants who are at higher risk of low birth weight and low nutritional reserves at birth [13]. Additionally, these risks can be made worse by inequalities in coverage of nutrition interventions and health care coverage. Equality across the socioeconomic spectrum is a positive force to enable households to obtain nutrient-rich foods and reduce health risks that are related to poor dietary practices and nutritional status; it is considered an essential component of the quality of health care [14,15].

Given the importance of women’s nutrition and health and dependence of nutrition interventions on health services, too little has been done to document the patterns of socioeconomic disparities and inequalities in women’s nutrition and health care coverage and access and how they differ across countries. A meta-analysis of Demographic and Health Survey (DHS) data from 31 countries documented the magnitude of associations between women’s health care utilization and wealth, education, and empowerment inequalities [16]. That study did not assess maternal nutrition interventions in health care, and the combined country estimates did not provide insights about country-specific differences. Inequalities in integrated nutrition and health services for pregnant and non-pregnant women have not been previously analyzed and highlighted in policy dialogue. A study that estimated inequalities in coverage of reproductive, maternal, newborn, and child health (RMNCH) services in 39 countries did not include nutritional status or nutrition services as one of the outcomes [17]. Individual country studies have documented the type and level of socioeconomic and rural/urban disparities but have not included inequalities in nutrition outcomes and health coverage and access [18–21]. Recent reviews of nutrition have focused more on aggregate trends [22,23], evidence of health impacts [24,25], sensitive and specific nutrition interventions [26], lack of service delivery indicators [27], and financing issues [28], while less attention was given to inequalities and the need to tailor interventions to reduce inequalities. The existing literature has the following gaps: inequality in health is reported, but there is a gap regarding inequality in nutrition, the studies did not explain the link between health services inequalities and nutrition inequalities, and there is a lack of emphasis on country-specific issues that is needed for developing effective and relevant solutions due to studies not reporting on country differences.

This study used nationally representative data from four high-burden countries in sub-Saharan Africa and South Asia to document several types of socioeconomic inequalities in health and nutrition indicators of adult women and the health-nutrition similarities. The outcomes selected for analysis were related to the findings in the literature on women’s nutrition practices, with a focus on health services that are critical for priority evidence-based nutrition interventions (for example, ANC visits are the main contact point for dietary counseling, for distributing supplements, and weight gain monitoring). They reflect Sustainable Development Goals and highlight nutrition factors that are key for health outcomes [2]. Factors influencing access to health care, such as perceived geographic proximity and need for permission to visit health facilities, are also included in this study [29,30].

## Methods

Bangladesh, Ethiopia, India, and Nigeria were selected as study countries since they are in the top tier of regions with the largest burdens of maternal mortality, malnutrition, and inadequate dietary intakes and contain diverse geographic regions with a diverse range of socioeconomic patterns and modes of health care delivery [1,17,18,25,31,32]. The countries are all located in the northern hemisphere and have varying altitudes that differ across areas, more so in India and Ethiopia than in Bangladesh and Nigeria. These differences could affect nutrition and health variables. Furthermore, Ethiopia recently faced drought and famine, socio-political disruptions, and reductions in foreign aid. Data for this study were obtained from the most recent comprehensive DHS conducted in Bangladesh (DHS 2017–18), Ethiopia (DHS 2016), India (DHS 2019–21), and Nigeria (DHS 2018). Precautions taken while collecting data during the SARS-CoV-2 included thermal screening, sanitizing hands and equipment, and using masks and gloves. The study population included all married and unmarried women in the age-group 20–49 years except in Bangladesh, where only married women were surveyed.

### Outcome indicators

Outcomes examined in this study include nutritional status, health services coverage, and health care access. Nutritional status indicators were low BMI (<18.5) sometimes referred to as thinness, and high BMI (BMI ≥ 25.0) sometimes referred to as overweight and obesity, and anaemia based on hemoglobin (Hb) levels (cut-off points used for anaemia were Hb < 11 gm/dL in pregnant and Hb < 12 gm/dL in non-pregnant women [33]. BMI was assessed only in non-pregnant women as the currently available global definitions are based on non-pregnant women only, while anaemia was assessed for all women using different definitions of anaemia (cut-off points) for pregnant and non-pregnant women. The respondents included women with a birth in the previous five years (or previous three years for Bangladesh).

Health services coverage was calculated for the most recently completed pregnancy in all women: i) IFA consumption (consumption of IFA tablets for 90 or more days during pregnancy), ii) four or more ANC visits [6], and iii) institutional delivery. Health care access was calculated for all women: i) distance to health facility as reported by respondents to be ‘a big problem’ and ii) needing permission from family members to go to a health facility as reported by respondents to be ‘a big problem’.

### Inequality

The study used Erreygers index (EI) that quantifies the existing inequality by assessing the distribution of a binary outcome of interest across the wealth spectrum (wealth inequality) or education spectrum (education inequality) [34]. EI ranges between −1 to +1 where 0 indicates no inequality, −1 indicates concentration of the outcome among the worse off (poor or uneducated), and +1 indicates concentration among the better off (higher wealth or more highly educated). For the wealth spectrum, the women’s households were ranked by a wealth index score that was calculated using principal component analysis of asset ownership and housing characteristics, as available within the DHS dataset. For the education spectrum, the number of years of completed schooling was used. The choice of socioeconomic measures was based on program and policy relevance for women’s nutrition and health and the data available in the DHS datasets. The terminologies of ‘disparities’ and ‘inequalities’ are often used interchangeably in the literature; in this paper, we use ‘disparities’ for socioeconomic status and ‘inequalities’ when referring to nutritional status and health care variables.

### Data analysis

The analysis involved calculating EI at the national level and in urban and rural populations for each country using the Stata ‘conindex’ command [35]. Tests were conducted for the EI scores and for the differences between urban and rural populations [36]. The hypothesis underlying tests for EI scores is that the index equals zero when there is perfect equality. The classification of survey clusters as urban or rural was defined by DHS for each country, primarily based on population density but also considering other criteria including percentage of population involved in agriculture, availability of electricity or piped water, and ease of access to healthcare, schools, or transportation [36]. All calculations accounted for the complex survey design of DHS and sampling weights.

## Results

The Ethiopian sample had a higher proportion of younger women as compared to the other countries (Table 1). Over half the women in Ethiopia had no education, while 47% to 61% of women in the other countries had completed secondary or higher education. Women in Ethiopia had lower access to media, lower access to improved water and sanitation facilities, and spent more time fetching water, suggesting higher daily energy expenditure. India had the lowest female labor force participation, and Nigeria the highest, with 42% in clerical/sales occupations. Bangladesh had the highest participation in agriculture (35%).

**Table 1.** Characteristics of the study population by country and survey year as weighted column percentages.

	Bangladesh 2017–18	Ethiopia 2016	India 2019–21	Nigeria 2018
Age-groups (n)	18,176	12,185	601,635	33,398
20–29 years	40	46	39	42
30–39 years	35	35	33	35
40–49 years	25	19	28	23
Marital status	18,175	12,185	601,635	33,398
Unmarried	0	11	11	12
Married	100	89	89	88
Education level (n)	18,176	12,185	601,635	33,398
No education	18	57	26	37
Primary	32	27	13	15
Secondary & higher	50	16	61	47
Place of residence (n)	18,176	12,185	601635	33398
Urban	29	22	33	46
Rural	71	78	67	54
Woman’s occupation	18,170	12,185	90,433	33,398
Not working	47	47	66	24
Professional/technical/managerial	2	3	4	7
Clerical/sales	2	17	3	42
Agricultural	35	22	15	16
Services/household and domestic	6	3	4	8
Skilled and unskilled manual	8	6	7	4
Communication channels (n)	18,175	12,185	601,635	33,398
Radio ≥ once a week	2	16	4	31
TV > once a week	55	15	54	33
Owns mobile telephone	61	27	58	60
Improved water source (n)	18,175	12,185	601,635	33,398
Unimproved water	2	34	4	26
Improved water	98	66	96	74
Improved sanitation (n)	18,175	12,185	601,635	33,398
Improved sanitation	50	7	71	34
Unimproved sanitation	49	62	11	43
Open defecation	1	31	18	22
Time to fetch water (n)	na	12,132	601,203	33,366
Half hour or less	na	72	99	93
More than half hour	na	28	1	7

n: Unweighted sample size; na: Information not available

The prevalence of low BMI was highest in Ethiopia at 20%, while that of anaemia was highest in Nigeria and India at 57% each (Table 2). Almost 30% or more women had high BMI in Bangladesh, India, and Nigeria. IFA consumption for at least 90 days during pregnancy was reported by around half of the women in each study country, except Ethiopia, where only 13% women reported consuming IFA for at least 90 days. Four or more ANC visits were reported by less than half the women in Bangladesh and Ethiopia, whereas almost six out of every ten women in India and Nigeria completed at least four visits. Institutional delivery was the lowest in Ethiopia at 31% and highest in India at 90%. Among the study countries, going to the health facility was considered a big problem for Ethiopian women both due to distance (51%) and needing permission to go (32%). Descriptive information on household and individual characteristics suggests a cluster of adverse conditions experienced by women in Ethiopia, such as low education levels and low exposure to media, inadequate water and sanitation facilities, low health coverage, and more geographic and social barriers for accessing health facilities. In Nigeria, there was a relatively high proportion of urban residents, more women with high BMI, a high prevalence of anaemia, and a lower prevalence of low BMI compared to other countries. In Nigeria, almost one in four women reported distance to facilities was a big problem, but needing permission to go was not.

**Table 2.** Prevalence of nutrition and health care indicators by country and survey year as weighted column percentages.

	Ethiopia 2016	India 2019–21	Nigeria 2018
Low BMI (n)	10,631	557,798	10,690
No	80	86	91
Yes	20	14	9
High BMI (n)	10,631	557,798	10,690
No	91	72	67
Yes	9	28	33
Anemia (n)	11,317	574,437	11,972
No	75	43	43
Yes	25	57	57
IFA supplements > 90 (n)	2,997	147,270	13,238
No	88	39	52
Yes	13	61	48
Number of ANC visits (n)	6,816	169,666	20,277
<4 visits	68	41	41
4 or more visits	32	59	59
Institutional delivery (n)	68,35	171,962	20,599
No	69	10	58
Yes	31	90	42
Distance to health facility (n)	12,185	601,635	33,398
Not a big problem	49	77	74
Big problem	51	23	26
Needing permission (n)	12,185	601,635	33,398
Not a big problem	68	87	89
Big problem	32	13	11

In all countries, wealth and education inequalities in low BMI favored the poor and less educated women, and the inequality was higher in rural areas as compared with urban areas (Table 3). Wealth-related inequality for high BMI favored the better-off women in all study countries. It was higher in urban areas of Bangladesh and Ethiopia and rural areas of India, with no difference in Nigeria. Inequality in high BMI was greater among the educated in all study countries. Wealth inequality in high BMI was not limited to urban areas in the study countries except in Ethiopia, and whereas education inequality in high BMI was higher in urban areas of Bangladesh and Ethiopia, it was higher in rural areas of India and Nigeria. Anaemia was concentrated at the lower end of the education and wealth spectrums at country level and in urban and rural areas.

**Table 3.** Inequalities in nutritional status (i.e. BMI for non-pregnant women only and anaemia for all women using different cut-off points for pregnant and non-pregnant women) estimated by Erreygers index by country and residence.

	Bangladesh	Ethiopia	India	Nigeria
	Low BMI – inequality by wealth index score			
Country	−0.133*	−0.088*	−0.139*	−0.103*
Urban	−0.096^*,#^	−0.001^#^	−0.072*^,#^	−0.052*^,#^
Rural	−0.138^*,#^	−0.054*^,#^	−0.129*^,#^	−0.111*^,#^
	Low BMI – inequality by years of education			
Country	−0.070*	−0.061*	−0.034*	−0.086*
Urban	−0.051*	0.023^#^	−0.003^*,#^	−0.036^*,#^
Rural	−0.070*	−0.034^*,#^	−0.018^*,#^	−0.087^*,#^
	High BMI – inequality by wealth index score			
Country	0.318*	0.181*	0.251*	0.362*
Urban	0.316^*,#^	0.300^*,#^	0.154^*,#^	0.287*
Rural	0.260^*,#^	0.030^*,#^	0.213^*,#^	0.295*
	High BMI – inequality by years of education			
Country	0.138*	0.119*	0.071*	0.257*
Urban	0.143^*,#^	0.089^*,#^	−0.013^*,#^	0.149^*,#^
Rural	0.109^*,#^	0.015^*,#^	0.052^*,#^	0.215^*,#^
	Anemia – inequality by wealth index score			
Country	na	−0.143*	−0.102*	−0.141*
Urban	na	0.013^#^	−0.080^*,#^	−0.124^*,#^
Rural	na	−0.134^*,#^	−0.092^*,#^	−0.091^*,#^
	Anemia – inequality by years of education			
Country	na	−0.093*	−0.068*	−0.123*
Urban	na	−0.040	−0.067^*,#^	−0.089*
Rural	na	−0.068*	−0.051^*,#^	−0.096*

Negative Erreygers index indicates higher concentration among the poor and less educated women. Positive Erreygers index indicates higher concentration among the non-poor and more educated women. *Erreygers index with *p* < 0.05. ^#^Urban and rural Erreygers index was different with *p* < 0.05. na: Information not available.

Economically well-off and better-educated women in the study countries had higher coverage of IFA consumption, four or more ANC visits, and institutional deliveries, whereas geographic and social constraints in accessing health facilities were concentrated among the poor and less-educated women (Table 4). Inequality in IFA consumption was greater in urban areas except in India where rural women showed higher inequalities by wealth and education. Education inequality in IFA was also higher in rural areas of Bangladesh. Compared with other countries, wealth and education inequality in IFA consumption was low in Ethiopia at country level and in rural areas, although wealth inequality was high in urban areas. As compared with rural areas, inequality in ≥4 ANC visits by wealth and education was greater in urban areas of Bangladesh and Ethiopia but lower in urban areas of India and Nigeria. Inequality in ≥4 ANC visits was high in Nigeria across wealth and education spectrum, reaching almost 0.50 (0.495 for wealth and 0.492 for education) at the country level. Coverage of institutional deliveries was concentrated among the economically better off and more educated women in all countries. In Nigeria, inequality in institutional delivery was high, with EI reaching almost 0.6 (0.573 for wealth, 0.583 for education). In Bangladesh as well, there was high inequality in institutional deliveries across the wealth (0.469) and education (0.425) spectrums. Inequality in access to health facilities was higher among the poorer and less educated women in all study countries, both in terms of geographic access/distance and in social access or needing permission to go. Inequalities in access to health facilities were highest in Ethiopia.

**Table 4.** Inequalities in health coverage and access estimated by Erreygers index by country and residence.

	Bangladesh	Ethiopia	India	Nigeria
**Coverage indicators (refers to the last completed pregnancy)**				
	≥90 days of IFA – inequality by wealth index score			
Country	0.210*	0.043	0.218*	0.230*
Urban	0.293^*,#^	0.135^*,#^	0.137*	0.187*
Rural	0.154	−0.003^#^	0.177*	0.178*
	≥90 IFA – inequality by years of education			
Country	0.297*	0.059*	0.206*	0.233*
Urban	0.279^*,#^	0.048	0.160*	0.192*
Rural	0.294^*,#^	0.040	0.183*	0.189*
	4 or more ANC visits – inequality by wealth index score			
Country	0.336*	0.282*	0.260*	0.495*
Urban	0.410^*,#^	0.383^*,#^	0.150^*,#^	0.301^*,#^
Rural	0.244^*,#^	0.152^*,#^	0.235^*,#^	0.395^*,#^
	4 or more ANC visits – inequality by years of education			
Country	0.322*	0.244*	0.236*	0.492*
Urban	0.360^*,#^	0.211^*,#^	0.162^*,#^	0.341^*,#^
Rural	0.281^*,#^	0.147^*,#^	0.222^*,#^	0.386^*,#^
	Institutional delivery – inequality by wealth index score			
Country	0.469*	0.410*	0.168*	0.573*
Urban	0.463^*,#^	0.348^*,#^	0.088^*,#^	0.398^*,#^
Rural	0.391^*,#^	0.185^*,#^	0.179^*,#^	0.414^*,#^
	Institutional delivery – inequality by years of education			
Country	0.425*	0.357*	0.167*	0.583*
Urban	0.434^*,#^	0.264^*,#^	0.104^*,#^	0.468^*,#^
Rural	0.393^*,#^	0.191^*,#^	0.175^*,#^	0.424^*,#^
**Access indicators**				
	Distance to health facility – inequality by wealth index score			
Country	−0.225*	−0.406*	−0.219*	−0.263*
Urban	−0.205^*,#^	−0.069^#^	−0.103^*,#^	−0.123^*,#^
Rural	−0.167^*,#^	−0.214^*,#^	−0.182^*,#^	−0.243^*,#^
	Distance to health facility – inequality by years of education			
Country	−0.155*	−0.238*	−0.147*	−0.182*
Urban	−0.141*	−0.030	−0.086^*,#^	−0.076^*,#^
Rural	−0.135*	−0.064*	−0.116^*,#^	−0.147^*,#^
	Needing permission to go – inequality by wealth index score			
Country	−0.047*	−0.205*	−0.095*	−0.080*
Urban	−0.066^*,#^	−0.049	−0.060^*,#^	−0.029^*,#^
Rural	−0.025^*,#^	−0.101*	−0.078^*,#^	−0.069^*,#^
	Needing permission to go – inequality by years of education			
Country	−0.043*	−0.147*	−0.062*	−0.083*
Urban	−0.058^*,#^	−0.009	−0.042^*,#^	−0.037^*,#^
Rural	−0.030^*,#^	−0.067*	−0.048^*,#^	−0.081^*,#^

Negative Erreygers index indicates higher concentration among the poor and less educated women. Positive Erreygers index indicates higher concentration among the non-poor and more educated women. *Erreygers index with *p* < 0.05. ^#^Urban and rural Erreygers index was different with *p* < 0.05.

## Discussion

This study describes both women’s nutritional status and components of health services that deliver women’s nutrition interventions and describes inequalities using nationally representative data from Bangladesh, Ethiopia, India, and Nigeria. While key indicators of nutritional status and health care adequacy were favorable for the better-off and more educated women in all countries, each country showed different magnitudes of inequalities and unique patterns of inequalities and clusters of nutrition and health-related challenges. Individual countries would benefit from documenting, developing tailored strategies, and monitoring their own patterns of inequality and coverage in women’s nutrition and health. For example, inequalities were higher in urban versus rural areas of Ethiopia across most indicators, whereas in other countries the concentration of inequalities by rural or urban residence differed by indicator. Since IFA supplements and counseling on IFA are usually provided during ANC visits, we expected inequality patterns to be similar for ANC and IFA supplements consumed for at least 90 days; the results show similarities but also differences.

Our findings are consistent with papers on the association between socioeconomic disparities and inequalities in women’s diets and nutrition, and use of health services [3,37,38]. In Nepal, despite an increase in ANC attendance and institutional delivery during 2001–2014, inequality in wealth and mothers’ education remained high with fewer women from low socioeconomic backgrounds attending ANC, delivering without skilled assistance, and not utilizing health and nutrition services [19]. A study in India found that urban residence, low education, and belonging to Scheduled Castes and Scheduled Tribes were associated with continued inequality in women’s health care [18]. In Bangladesh, a decreasing trend in wealth inequality in ANC services, health facility delivery, and skilled birth assistance was found in urban but less so in rural areas, and coverage was higher among the better-off [39,40]. In another study, women in Bangladesh from the poorest wealth quintile had the highest odds of being undernourished, while education and media access were associated with better nutrition [41]. In Sierra Leone, while inequalities in ANC coverage had narrowed during 2008–2019, they still remained for women’s health outcomes and skilled birth attendance, favoring the wealthy, more educated, and urban groups [21]. In a study of 366 districts in Kenya, Uganda, and Tanzania, several districts showed lower coverage of four or more ANC visits among the poor, uneducated, and those geographically marginalized from healthcare [20].

Tailoring strategies to remedy factors underlying country-specific inequalities requires participatory co-designing of relevant interventions and program service delivery options jointly with affected populations, disaggregated by urban and rural settings. For addressing nutrition, epidemiologic data show that diet quality follows a socioeconomic gradient, and explanatory factors are likely to involve several underlying behavioral factors [42]. The recommended interventions involve a combination of taxes and strong regulations to discourage intakes of unhealthy foods, complemented by targeted approaches to reach underprivileged women with a package of nutrition and health care interventions through local food markets and health services and multiple information channels [25,43–45]. A decomposition analysis of inequality in Ethiopia identified dual factors of education and access to information through media as well as wealth as equally important in motivating and enabling women to access life-saving health services [46]. Differential patterns of inequality trends are illustrated in a study from Uganda where inequalities expanded over time in the prevalence of anaemia and low BMI while healthcare utilization for skilled birth attendants and facility delivery showed less inequality in successive DHS surveys [47]. This suggests that factors outside the health system are important, and investments are needed for better data on underlying drivers of inequalities, for monitoring nutrition and related health outcomes, and to understand predisposing factors. WHO’s recommendations for improving ANC services included guidance on the provision of outreach and community-based services during pregnancy [6]. Targeting of interventions with proven benefits for undernourished women based on rapid screening methods was recommended previously [2,48]. A review of the impact of dietary behavior modification interventions to prevent NCDs and to assess health equity impacts found that policies to reduce economic barriers appeared likely to decrease health inequalities across socioeconomic groups, whereas interpersonal counseling interventions that relied on frequent contact with formal health services did not [49].

Our study used cross-sectional data and so causality cannot be assumed. Also, DHS surveys do not provide a full set of variables for characterizing nutrition interventions for women; for example, information is not available on diets, nutrient content of dietary intakes, dietary counseling, and gestational weight gain during ANC. Nevertheless, our study adds important findings to the existing body of literature using available datasets.

## Conclusions

Substantial wealth- and education-related inequalities were found in nutrition and related health services in four high burden LMICs and favored better-off and more educated women. Inequalities across countries differed by urban/rural residence and type of indicator. Targeting nutrition and related health services use and access by those most affected should be the focus of governments. This would help in reducing inequalities which would in turn help in attaining the SDGs faster. The different patterns of nutrition, health coverage and access, and inequalities across the four LMICs suggest that countries need to understand their unique patterns of inequalities in women’s nutritional status and health care.

## Data Availability

No new primary data were collected. A secondary analysis of DHS data was conducted. Deidentified participant data are available from DHS by writing to archive@dhsprogram.com
